# National genomic epidemiology investigation revealed the spread of carbapenem-resistant *Escherichia coli* in healthy populations and the impact on public health

**DOI:** 10.1186/s13073-024-01310-x

**Published:** 2024-04-16

**Authors:** Yan Li, Yanyan Zhang, Xinran Sun, Yuchen Wu, Zelin Yan, Xiaoyang Ju, Yonglu Huang, Hongwei Zhou, Zhiqiang Wang, Shaolin Wang, Rong Zhang, Ruichao Li

**Affiliations:** 1 Jiangsu Co-Innovation Center for Prevention and Control of Important Animal Infectious Diseases and Zoonoses, College of Veterinary Medicine, Yangzhou University, 48 East Wenhui Road, Yangzhou, Jiangsu 225009 P. R. China; 2 Department of Clinical Laboratory, Second Affiliated Hospital of Zhejiang University, School of Medicine, Hangzhou, Zhejiang P. R. China; 3 College of Veterinary Medicine, China Agricultural University, Beijing, P. R. China; 4 Jiangsu Key Lab of Zoonosis, Yangzhou University, Yangzhou, Jiangsu P. R. China; 5 Institute of Comparative Medicine, Yangzhou University, Yangzhou, Jiangsu P. R. China

**Keywords:** Healthy populations, Carbapenem resistance, *E. coli*, Plasmids, Diverse clones

## Abstract

**Background:**

Carbapenem-resistant *Escherichia coli* (CREC) has been considered as WHO priority pathogens, causing a great public health concern globally. While CREC from patients has been thoroughly investigated, the prevalence and underlying risks of CREC in healthy populations have been overlooked. Systematic research on the prevalence of CREC in healthy individuals was conducted here. We aimed to characterize CREC collected from healthy populations in China between 2020 and 2022 and to compare the genomes of CREC isolates isolated from healthy individuals and clinical patients.

**Methods:**

We present a nationwide investigation of CREC isolates among healthy populations in China, employing robust molecular and genomic analyses. Antimicrobial susceptibility testing, whole-genome sequencing, and bioinformatics were utilized to analyze a cohort of CREC isolates (*n* = 113) obtained from fecal samples of 5 064 healthy individuals. Representative plasmids were extracted for third-generation nanopore sequencing. We previously collected 113 non-duplicate CREC isolates (59 in 2018, 54 in 2020) collected from ICU patients in 15 provinces and municipalities in China, and these clinical isolates were used to compare with the isolates in this study. Furthermore, we employ comparative genomics approaches to elucidate molecular variations and potential correlations between clinical and non-clinical CREC isolates.

**Results:**

A total of 147 CREC isolates were identified from 5 064 samples collected across 11 provinces in China. These isolates were classified into 64 known sequence types (STs), but no dominant STs were observed. In total, seven carbapenemase genes were detected with *bla*_NDM-5_ (*n* = 116) being the most prevalent one. Genetic environments and plasmid backbones of *bla*_NDM_ were conserved in CREC isolated from healthy individuals. Furthermore, we compared clinical and healthy human-originated CRECs, revealing noteworthy distinctions in 23 resistance genes, including *bla*_NDM-1_, *bla*_NDM-5_, and *bla*_KPC_ (*χ*^2^ test, *p* < 0.05). Clinical isolates contained more virulence factors associated with iron uptake, adhesion, and invasion than those obtained from healthy individuals. Notably, CREC isolates generally found healthy people are detected in hospitalized patients.

**Conclusions:**

Our findings underscore the significance of healthy populations-derived CRECs as a crucial reservoir of antibiotic resistance genes (ARGs). This highlights the need for ongoing monitoring of CREC isolates in healthy populations to accurately assess the potential risks posed by clinical CREC isolates.

**Supplementary Information:**

The online version contains supplementary material available at 10.1186/s13073-024-01310-x.

## Background

Carbapenems, which are considered the last line of defense, are frequently employed for the treatment of infections caused by multidrug-resistant pathogens [1]. However, carbapenem-resistant Enterobacteriaceae (CRE) are spreading at an alarming rate worldwide which has led to a severe public health issue [2]. Among them, *Klebsiella pneumoniae* and *E. coli* stand out as the most prevalent and deleterious [3]. CRE can induce severe infections such as urinary tract infections, bloodstream infections, and pneumonia. The emergence and spread of CRE have significantly limited therapeutic options, resulting in elevated rates of morbidity and mortality [4].

The increasing prevalence of carbapenem-resistant *Escherichia coli* (CREC) in hospitals and other healthcare facilities has raised alarms worldwide [5]. The emergence and spread of CREC primarily result from the acquisition of carbapenemase genes, such as the *bla*_KPC_, *bla*_NDM_, and *bla*_OXA-48_-like resistance genes. CREC has the potential to spread rapidly within healthcare facilities, particularly in intensive care units (ICUs) [6]. The rapid increase in CREC infections in the clinical system has become a serious challenge for healthcare systems, especially in low- and middle-income countries with limited resources for infection control and surveillance [7]. Furthermore, CREC can persist in the hospital environment, specifically on contaminated surfaces and medical equipment, facilitating transmission between patients and healthcare workers [8].

Although CREC has traditionally been recognized as a significant issue in healthcare settings, recent studies have revealed their prevalence in non-clinical environments such as sewage, soils, surface waters, industrial effluents, and vegetation [9, 10]. The spread of CREC in these settings is a growing concern due to the increased risk of transmission to vulnerable populations and the potential emergence of new antibiotic resistance genes (ARGs) [11]. Furthermore, research on hospital wastewater has demonstrated that CREC from hospital environments can be transmitted to other environments, posing a significant threat to public health [12].

Although previous studies have shed light on the prevalence of CREC in hospitals, community settings, and the environment, there remains a gap in our understanding of its prevalence among healthy populations [13]. Specifically, the diversity, persistence, transmission, and evolution of CREC within fecal microbiota in healthy individuals has not been comprehensively deciphered. To address this knowledge gap, we conducted a first national surveillance study between 2020 and 2022 to identify CREC isolates carried by healthy people in China. Using advanced genomic epidemiological analysis methods, we investigated the genomic characteristics of these isolates and highlight the potential risk they pose to human health. Our findings underscore the necessity for continuous surveillance of CREC in healthy populations and targeted control measures to mitigate this overlooked threat.

## Methods

### Study design

To investigate the prevalence of CREC in healthy populations, we consecutively collected 5064 non-duplicate stool specimens from healthy individuals without evidence of intestinal infection from hospitals across 11 regions in China between 2020 and 2022. These healthy individuals had come to the hospital for a routine physical examination. After incubating the samples in 5 mL Luria–Bertani broth at 37 °C for 6 h, the enriched cultures were screened for CREC isolates by streaking on MacConkey agar plates supplemented with meropenem (0.3 mg/L), and the species of isolates were identified by using MALDI-TOF MS. To avoid duplication, only one CREC isolate per stool sample was selected for further study. Ethical approval for this study was given by the Zhejiang University ethics committee (number 2023–0733). Antimicrobial susceptibility testing (AST) was performed on all non-duplicated isolates using the broth microdilution method against a panel of fifteen antimicrobials, with *E. coli* ATCC 25922 as the quality control. The resistance breakpoint of tigecycline (> 2 mg/L) was interpreted based on European Committee on Antimicrobial Susceptibility Testing (EUCAST) criteria (https://www.eucast.org/), and the Clinical and Laboratory Standards Institute (CLSI) guidelines were followed for the remaining fourteen antimicrobials [14].

### DNA extraction, whole-genome sequencing, and de novo assembly

To further investigate the genetic characteristics of CREC isolates, the genomic DNA of all CREC isolates were extracted using the PureLink Genomic DNA Mini Kit (Invitrogen, USA). Then, the high-quality genomes were subjected to whole-genome sequencing by the Illumina Hiseq 2500 platform with 2 × 150 bp paired-end libraries. In addition, plasmids of the representative CREC isolates were extracted for third-generation nanopore sequencing to obtain complete genome sequences. The plasmids of the isolates were extracted using the Qiagen plasmid midi-kit (Qiagen, Germany) after overnight culture in 100 ml LB broth. After performing quality control and filtering by fastqc, sequencing data of Illumina and nanopore were underwent separate assembly processes using SPAdes v.3.11.0 and Flye v.2.4.2, respectively [15, 16]. The plasmid sequences were completed with a hybrid de novo assembly strategy by Unicycler v.0.4.8 [17]. Genome assemblies were further confirmed by comparing the two assembly strategies.

#### Genomic analysis

The ARGs, insertion sequences, and plasmid replicons were detected using the online tools with default parameters [18–20]. Virulence factors (VFs) were determined using the virulence factor database (last updated 14 October 2020) in ABRicate [21]. Multiple sequence typing (MLST) and serotyping were performed using mlst v.2.15.1 and ECTyper v.1.0 with default parameters, respectively [19, 22]. The phylotyping of *E. coli* was performed using ClermonTyping software [23]. EasyFig v.2.2.3 and Brig v.0.95 was used to generate plasmid comparison figures  [24, 25]. The single nucleotide polymorphisms (SNPs) alignments were performed using Snippy v.4.0.2 [26].

### Phylogenetic and genome wide association analyses

Pan-genome and core-genome analyses were performed using Roary to determine the genetic diversity and relatedness [27]. The phylogenetic tree was generated by maximum likelihood method using FastTree, and the evolutionary relationships were visualized using iTOL [28, 29]. In addition, to further explore the genomic similarities and differences of CRECs between clinical patients and healthy people, 113 CREC isolates that we had previously isolated from ICU wards were included in this study for analysis [6]. The CREC isolates were divided into healthy people group and patient group. Scoary was employed for downstream whole-genome association analysis (GWAS) of CRECs for these two groups using the presence/absence matrix output generated by Roary as input files, to retrieve the different characteristics of both groups [30]. To annotate the functions of genomes, functional annotation of differentially expressed genes was determined based on the prokka v.1.12 alignment output [31]. The latest update database of eggNOG was used for function annotations and the annotations were further analyzed for KEGG pathway enrichment analysis [32, 33].

### Statistical analysis

Statistical analysis and visualization charts were performed using R v.4.2.2 (R Foundation for Statistical Computing, Vienna, Austria). The association between ARGs and insertion sequences was analyzed based on Spearman algorithm and visualized using Gephi [34]. Chi-square analysis was used to detect differentially expressed genes between healthy individuals and patients-derived CRECs. Principal coordinate analysis (PCoA) based on Bray–Curtis distance was performed to evaluate the composition of the VFs and ARGs in the CRECs isolated from different sources.

## Results

### Distribution and carbapenemase genes of CREC isolates among healthy populations

**Fig. 1:**
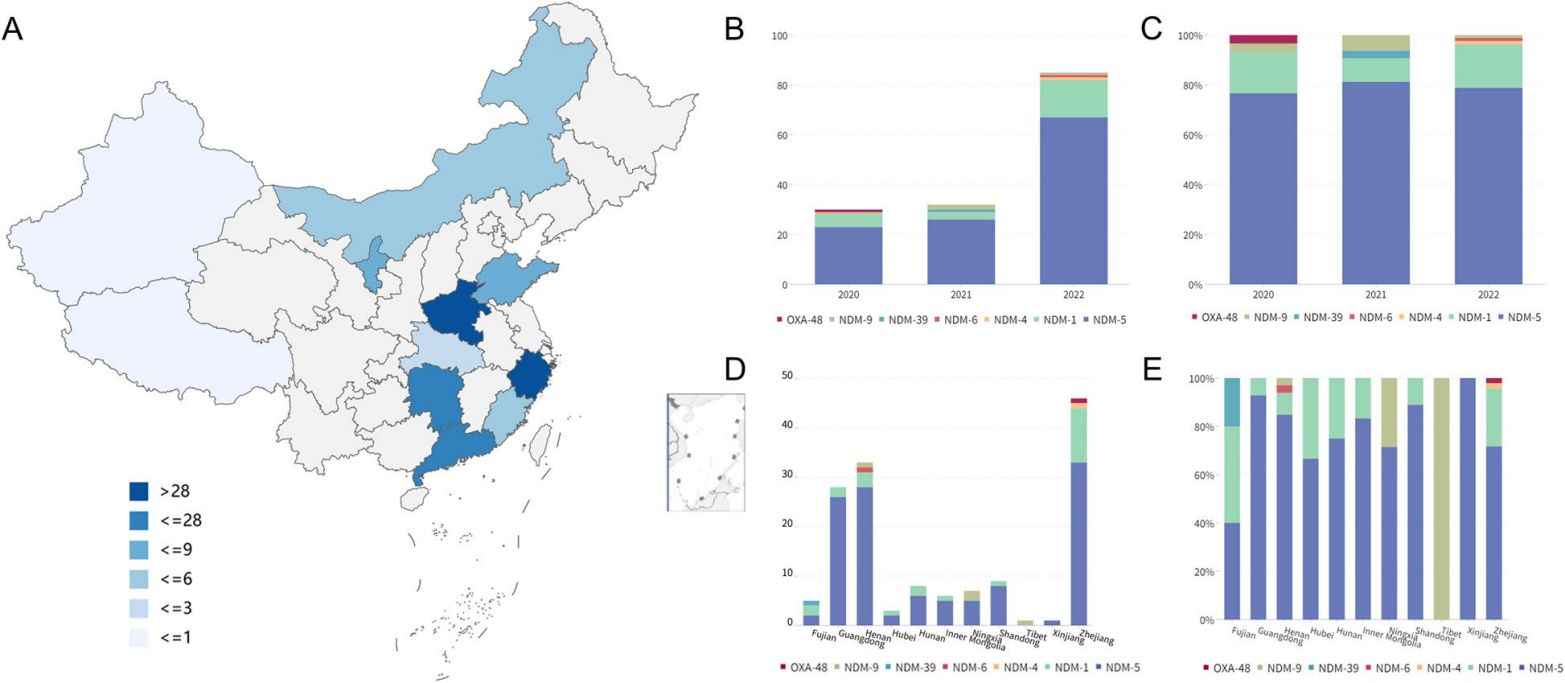
Distribution and prevalence of CRECs and corresponding carbapenemases in China. **A** Prevalence of CRECs in healthy individuals in China. Different colors represent the number of CRECs. **B** Bar graph showing the number of carbapenemases by year. **C** Bar graph displaying the percentage of different carbapenemases by year. **D** Bar graph displaying the number of different carbapenemases by regions. **E** Bar graph displaying the percentage of different carbapenemases by regions

In this study, we conducted a nationwide survey and isolated a total of 147 CRECs from March 2020 to September 2022. These isolates were obtained from 11 provinces in China, including Zhejiang, Guangdong, Henan, Xinjiang, Inner Mongolia, Tibet, Shandong, Hunan, Hubei, Ningxia, and Fujian (Fig. 1A, Table S1). Notably, all isolates were derived from stool samples of apparently healthy individuals, accounting for 2.90% of the total screened samples (147/5064). The geographical distribution of the samples covered a substantial portion of China, with a population of approximately 619 million individuals. This comprehensive sampling approach provides valuable insights into the prevalence and distribution of CREC isolates across different regions in China.

Among these CREC isolates collected from healthy individuals, a total of seven carbapenemase genes, including *bla*_NDM-1_, *bla*_NDM-4_, *bla*_NDM-5_, *bla*_NDM-6_, *bla*_NDM-9_, *bla*_NDM-39_, and *bla*_OXA-48_, were detected (Fig. 1B–E). Notably, the *bla*_NDM-5_ gene was the most prevalent carbapenemase gene (78.91%, 116/147), followed by the *bla*_NDM-1_ gene (15.65%, 23/147). Four isolates were found to carry the *bla*_NDM-9_ gene. However, the *bla*_NDM-6,_ *bla*_NDM-4_, *bla*_NDM-39_, and *bla*_OXA-48_ genes were each detected in only one isolate, respectively. The number of the *bla*_NDM_ variants in the CRECs increased gradually over time, with the *bla*_NDM-5_ gene dominated among the carbapenemase genes in different years. Furthermore, *bla*_NDM-5_ was the dominant carbapenemase gene in all regions except Fujian and Tibet.

### Antimicrobial susceptibility testing and ARGs diversity of CREC isolates

Among the tested carbapenems, CREC isolates showed the highest resistance rate to ertapenem (93.20%), followed by meropenem (92.52%) and imipenem (89.12%). All CREC isolates showed high-level resistance to the third- or fourth-generation cephalosporins and their combination with β-lactam inhibitors (91.84–100%). However, a limited number of isolates exhibited resistance to colistin (3.40%), tigecycline (4.08%), and amikacin (11.56%) (Table S2). The phenotype could in most cases be explained by carriage of the corresponding ARGs. Other than carbapenemase genes, CREC isolates also carry multiple ARGs including genes conferring resistance to beta-lactams (*bla*_CTX-M_ *n* = 49, *bla*_TEM_ *n* = 87, and *bla*_SHV_ *n* = 67), sulfonamide (*sul1 n* = 58, *sul2 n* = 70, *sul3 n* = 67), aminoglycoside (*aac*(3)*-II n* = 31, *aac(3)-Iva n* = 55, *aadA n* = 147, *rmtB n* = 15), and tetracycline (*tet*(M) *n* = 28). In addition, twelve and two CRECs were found to carry the *mcr-1* gene and the *tet*(X4) gene, respectively.

To explore the relationship between carbapenemase genes, other ARGs, and insertion sequences, a correlation network graph was constructed (Fig. S1). The network graph revealed strong associations between certain genetic elements. In particular, IS*21*, IS*Ec10*, IS*Ec20*, *bla*_CTX-M-15_, *bla*_EC-5_, *bla*_OXA-1_, and IS*Ec24* were strongly positively associated with *bla*_OXA-48_ (*R* > 0.3, *p* < 0.05). Similarly, IS*414* was positively associated with *bla*_NDM-1_ (*R* > 0.3, *p* < 0.05). IS*Ec46* and IS*903* were positively associated with *bla*_NDM-4_ (*R* > 0.3, *p* < 0.05). The *bla*_NDM-5_ gene was positively correlated with IS*10R* but negatively correlated with *bla*_NDM-1_ (*R* absolute value > 0.3, *p* < 0.05). *bla*_CTX-M-65_ was positive associated with *bla*_NDM-6_ (*R* > 0.3, *p* < 0.05). In addition, IS*1326*, IS*1353*, IS*10R*, *bla*_CTX-M-65_, *aac(6')-II*, and *aadA22* showed positive associations with *bla*_NDM-9_ (*R* > 0.3, *p* < 0.05).

### Transmission of CREC multiple clones

**Fig. 2:**
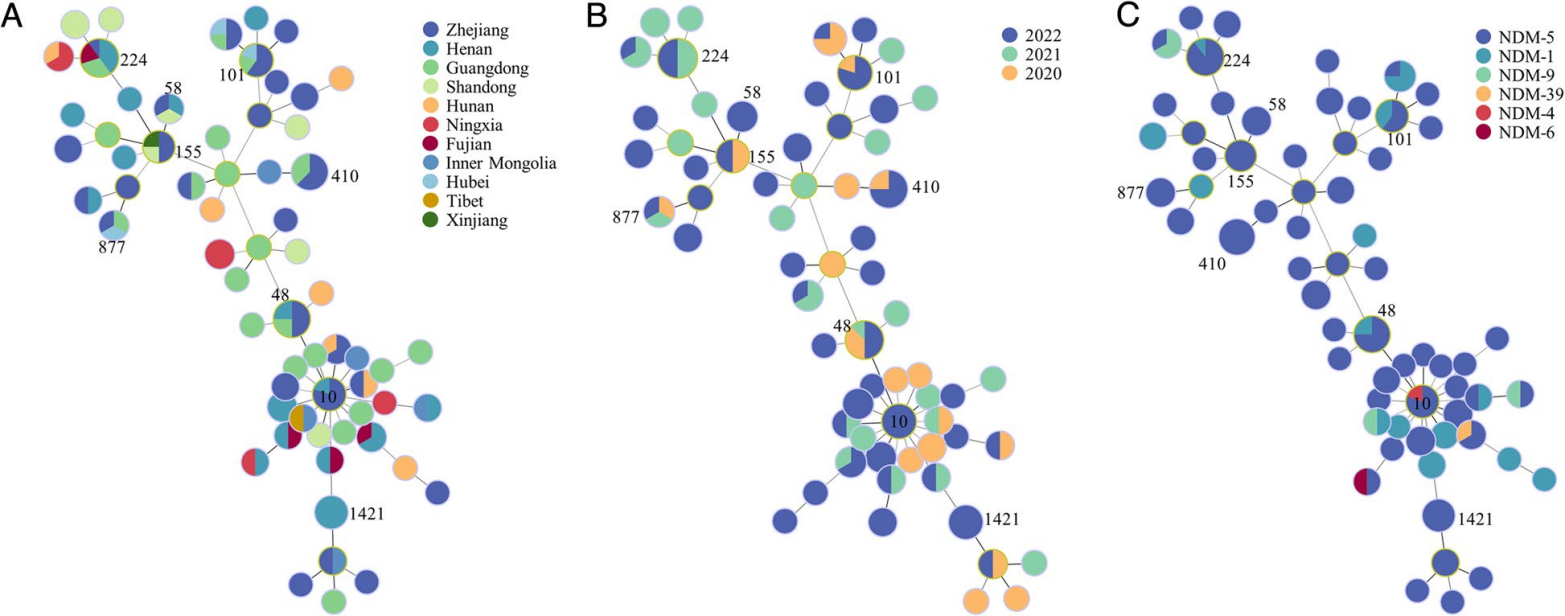
Minimal spanning tree of CREC isolates. **A** STs distribution of CREC isolates in different regions. **B** STs distribution of CREC isolates in different years. **C** STs distribution of different carbapenemases

The 147 CREC isolates belonged to 64 known STs, none of which exhibited a dominant prevalence. Our subsequent analysis revealed a significant correlation between the distribution of some STs and the geographical locations of the isolates, as demonstrated by the patterns depicted in Fig. 2A. Among the 64 STs, ST224 was the most widely dispersed ST, which was detected in five provinces and exhibited a relatively high isolation rate (10/147, 6.80%). Moreover, ST224 was also the predominant ST of the *mcr-1*-positive CRECs in this study. In terms of time distribution, only ST48 CREC was detected in all three years (Fig. 2B). The number of STs has been increasing over time. In addition, we found that some STs showed a corresponding relationship with carbapenem resistance genes. All ST410 and ST155 isolates were positive for *bla*_NDM-5_ (Fig. 2C). The *bla*_NDM-4_, *bla*_NDM-6_, and *bla*_NDM-39_ genes were only found in ST10, ST361, and ST746 isolates, respectively.

**Fig. 3:**
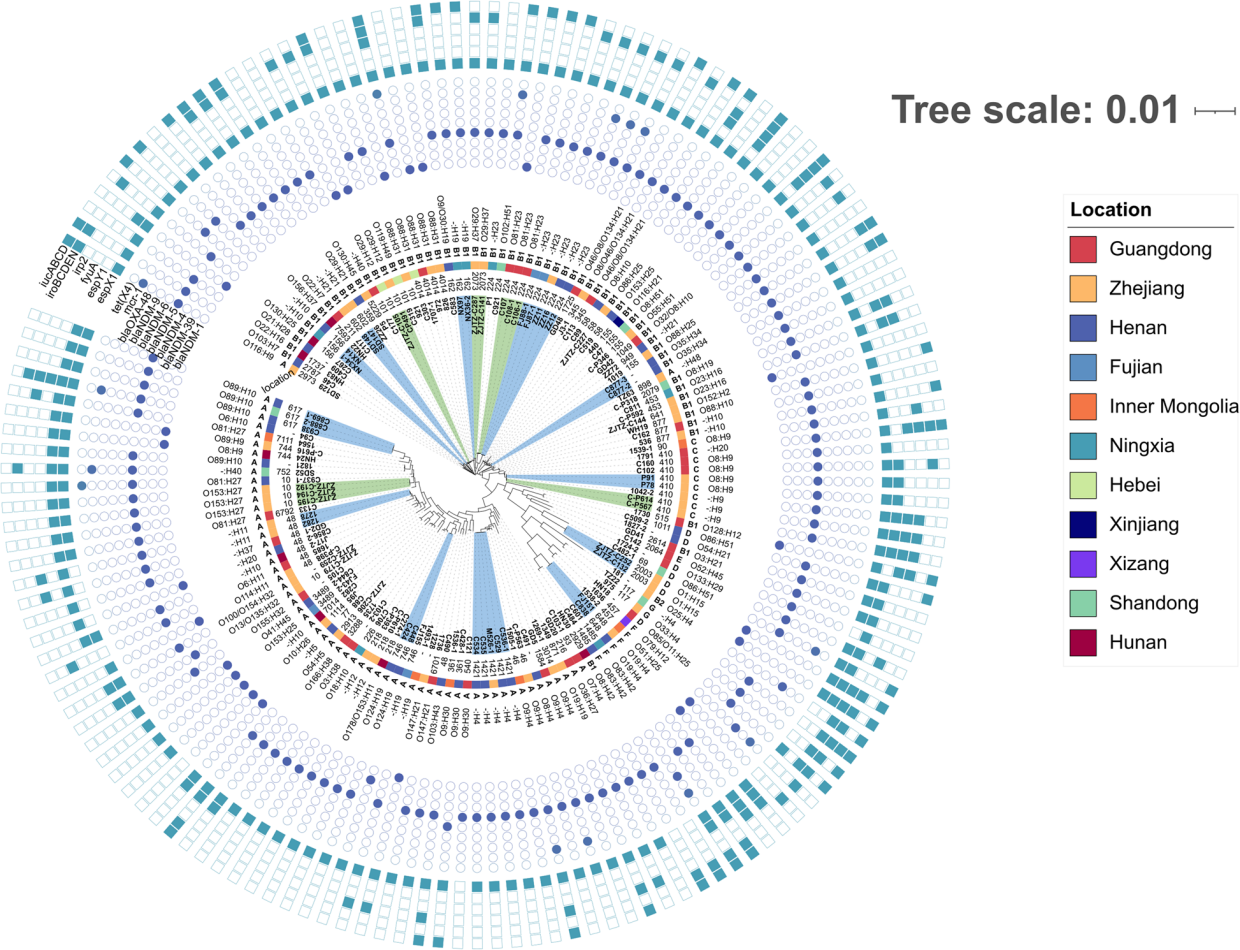
Phylogenetic tree of 147 CREC isolates from China. Isolates located in the light green or light blue shaded areas contained only a few SNPs differences (*n* ≤ 20). The resistance genes are indicated by square, solid graphics indicate yes, hollow no

The classification of phylogenetic subgroups demonstrated that the 147 CREC isolates were distributed into 7 phylogroups (A, B1, B2, C, D, F, and G), but the majority of them were in group A (61/147, 41.50%) and B1 (60/147, 40.81%) (Fig. 3). Serotype prediction revealed that there was no dominant serotype among the CREC isolates, and the most frequently isolated serotypes were O8:H9 (*n* = 6) and O88:H31 (*n* = 5). Furthermore, we observed a pronounced genetic resemblance among strains situated within the same clusters in the shadow, characterized by a high degree of similarity (≤ 20 SNPs), suggestive of clonal transmission. Subsequent analyses identified a total of 19 such clusters, with strains distributed across diverse STs, all of which exhibited multidrug resistance.

### Conserved genetic contexts of carbapenemase genes

The genetic contexts of the *bla*_NDM_ genes were observed to be highly conserved. A total of two major genetic contexts were identified in 116 *bla*_NDM-5_-positive isolates, including IS*3000-*ΔIS*Aba125-*IS*5-bla*_NDM-5_*-ble-trpF* and IS*3000-*IS*Aba125-*IS*5-bla*_NDM-5_*-ble-trpF*. The *bla*_NDM-5_ gene was predominantly carried by either IncX3 or IncHI2A plasmids (Fig. S2, Table S3). Besides, only one genetic context IS*3000-*IS*Aba125-bla*_NDM-1_*-ble-trpF-*IS*26* were identified in *bla*_NDM-1_-positive CREC isolates. All *bla*_NDM-1_-positive plasmids belonged to IncX3. All *bla*_NDM-9_-positive plasmids were of the IncHI2 type (4/4, 100%), and their conserved genetic context was ΔIS*Aba125-bla*_NDM-9_*-ble-trpF*. In addition, the genetic context of only identified *bla*_NDM-4_-positive isolate was IS*3000-*ΔIS*Aba125-bla*_NDM-4_*-ble-trpF*.

According to the core genome diversity and plasmid diversity comparison, all *bla*_NDM_-positive plasmids have a relatively conservative backbone structure. Besides, most plasmids showed no significant correlation with bacteria evolution (Fig. S3), implying conserved plasmids contributed to the spread of *bla*_NDM_ in CREC isolates. However, there were also some isolates with a closer evolutionary relationship and higher homology of *bla*_NDM_-positive plasmids, indicating that clone transmission of *bla*_NDM_-positive isolates also plays a role in the spread of CREC isolates.

### Different phylogenetic evolution of CRECs from clinical and healthy populations

**Fig. 4:**
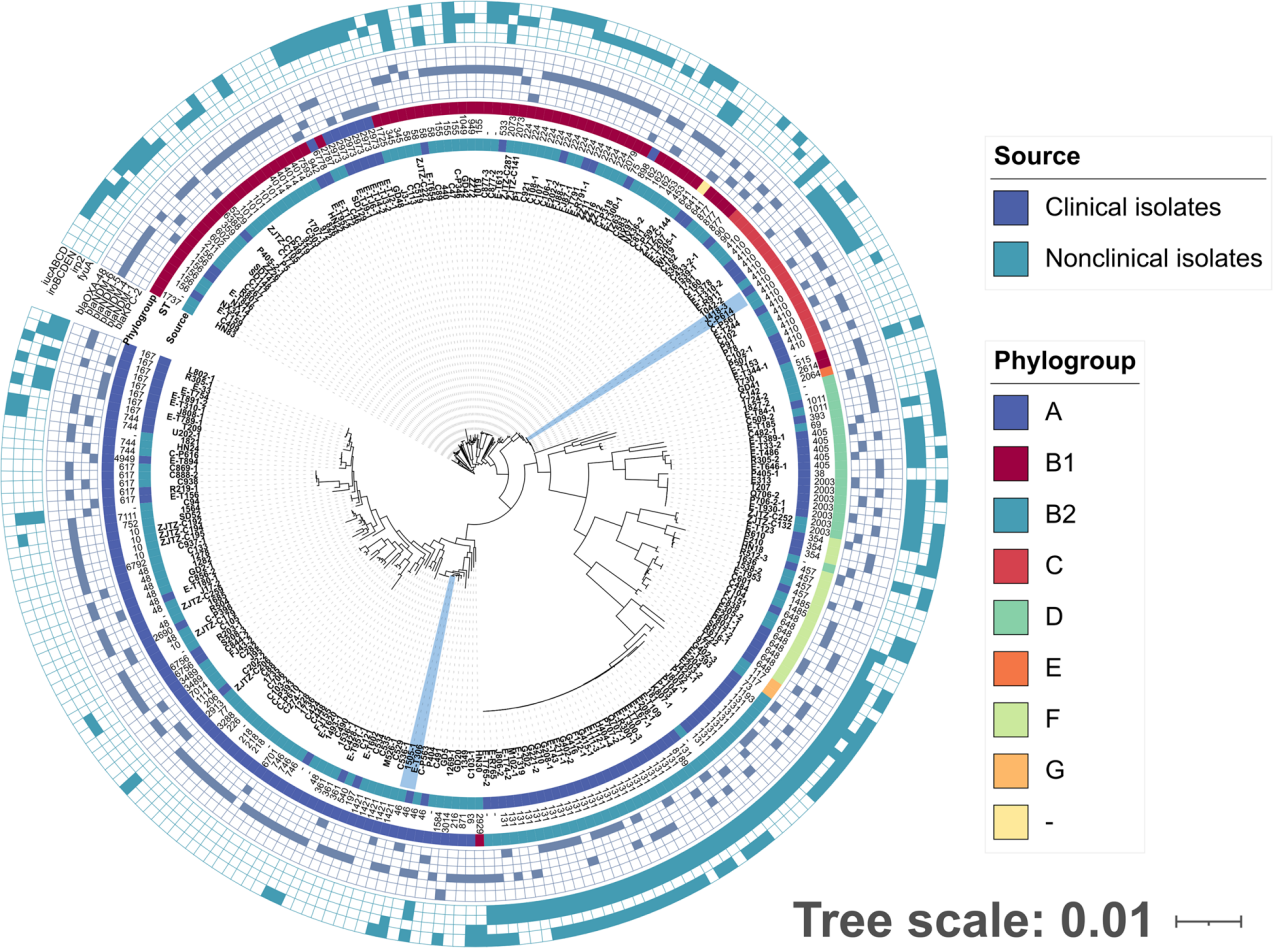
Phylogenetic tree of 260 CREC isolates from clinical and non-clinical environments. Clinical and non-clinical CRECs have different genetic backgrounds, with significant differences between their STs, ARGs, and phylogroup. The resistance genes are indicated by square, solid graphics indicate yes, hollow no. The isolates in the blue-shaded areas represent isolates from different sources but with high genetic similarity (≤ 20 SNPs)

In order to further describe the genetic features of CRECs in healthy populations, 113 CREC isolates of clinical origins were downloaded from the NCBI database [6]. These 113 CREC isolates were collected in our previous study from ICU patients in 15 provinces and municipalities in China. Among the 260 CREC isolates, we detected 100 known STs, with a marked difference in ST distribution between clinical and non-clinical *E. coli* isolates (Fig. 4). Specifically, the CRECs isolated from healthy individuals exhibited a higher diversity of STs (*n* = 48) compared to clinical CRECs (*n* = 20). The two groups shared only 16 STs (16/100, 16%). ST131 was the most prevalent (34/113,30.09%) in clinical CRECs, while ST224 being the most common in non-clinical CRECs (10/147, 6.8%). The distribution of *bla*_KPC_-positive isolates was only observed in clinical samples, with ST131 carrying a larger number of the *bla*_KPC_ genes. The isolates located in the shaded areas of Fig. 4 are those from different sources but with high genetic similarity (≤ 20 SNPs), indicating the possibility of clonal transmission between nonclinical and clinical CRECs (Table S4). The STs of these isolates were ST46 and ST410, distributed across various regions (Shanghai, Zhejiang, Inner Mongolia), forming two distinct clusters of distribution, and all of them carried carbapenemase genes.

### Genomic differences in CREC isolates in clinical and non-clinical settings

**Fig. 5:**
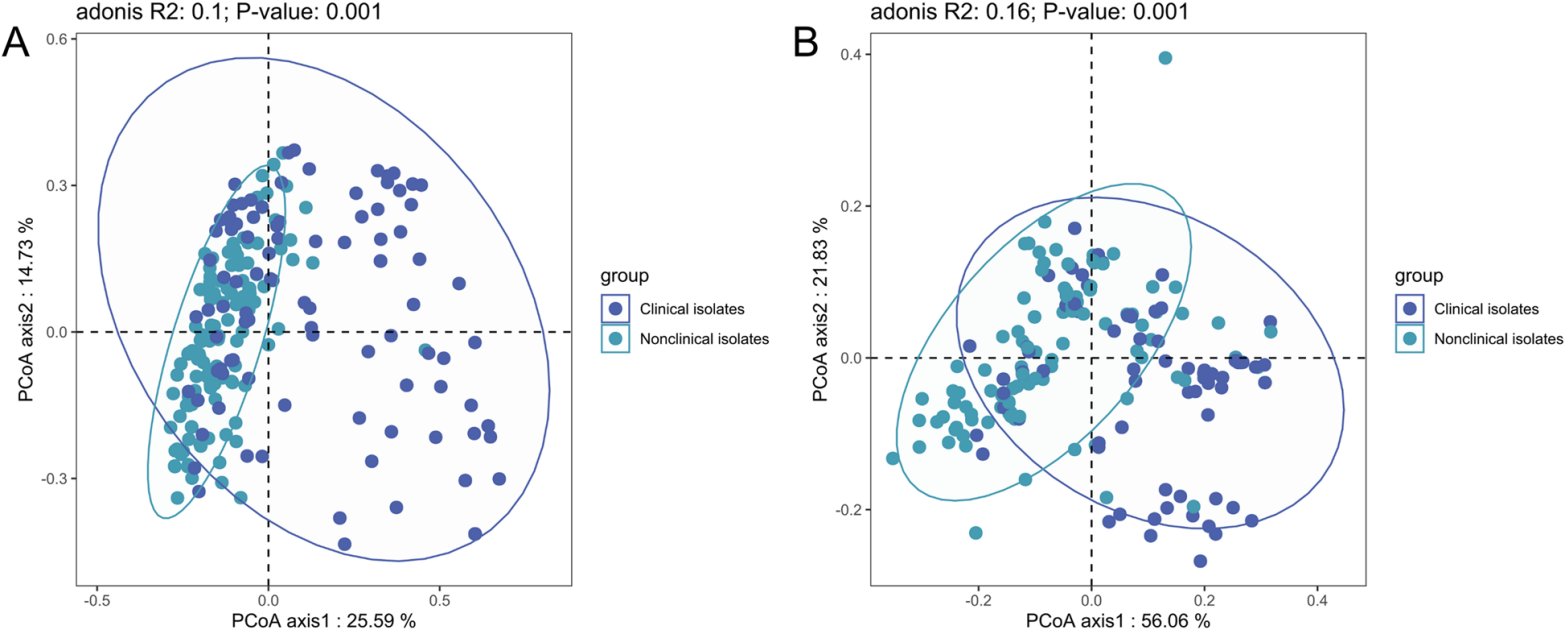
Principal component analysis for ARGs and VFs. **A** Principal component analysis of ARGs in CRECs from healthy people and clinical sources. **B** Principal component analysis of VFs in CRECs from healthy people and clinical sources

Compared with 113 clinical isolates, the CREC isolates from healthy individuals carried a greater variety of insertion sequences and VFs. However, CREC from healthy individuals carries fewer types of ARGs and plasmid replicons (Fig. S4). The positive rate of the *intI1* gene in clinical isolates was 74.34% (84/113), and in isolates collected from healthy individuals, it was 75.51% (111/147). Alpha diversity analysis showed that compared with the CRECs from healthy people, the CRECs from clinical sources carried more plasmid replicons and VFs (*p* < 0.05). However, there was no significant difference in the number of resistance genes and insertion sequences between these two sources of CRECs (*p* > 0.05) (Fig. S5). PCoA results showed that compared with the CRECs isolated from healthy people, the ARGs and VFs carried by different CREC isolates isolated from clinical samples varied greatly (Fig. 5).

Among all 120 detected ARGs, 23 ARGs exhibited significant differences between healthy and clinical population groups (*χ*^2^ test, *p* < 0.05) (Table S5, Fig. S6A). Besides, significant differences were observed in the positive rates of carbapenemase genes between clinical and non-clinical isolates, with *bla*_NDM-5_ being more prevalent in non-clinical isolates (*χ*^2^ test, *p* < 0.01) and *bla*_KPC-2_ being more prevalent in clinical isolates (*χ*^2^ test, *p* < 0.01). In non-clinical CREC isolates, the enriched ARGs for aminoglycosides included *aac*(3)*-IVa*, *aadA*, *aph*(3')*-Ia*, *aph*(4)*-Ia*, *arr-2*, and *dfrA17*, while the positive rates of resistance genes for β-lactams and quinolones were significantly higher than those of clinical CREC isolates.

A total of 65 VFs that showed significant differences between the two groups were significantly more abundant in clinical isolates (*χ*^2^ test, *p* < 0.01) (Table S6, Fig. S6B). We found that the positive rates of several important virulence genes *fyuA*, *irp2*, and *iucABCD* in clinical isolates (57.52%, 57.22%, 53.10%) were higher than those in isolates isolated from healthy individuals (12.93%, 11.56%, 30.61%). All VFs with significantly higher prevalence are rich in clinical CREC, which indicated that these clinical CREC isolates have higher pathogenicity. Further looking up VFs against VFDB, these differentially abundant VFs were mainly associated with invasion (e.g., K1 capsule), iron uptake (e.g., ferrienterochelin receptor Fes), secretion (e.g., type II secretion system, type III secretion system), and adhesion (e.g., fimbrial protein). Those virulence factors are crucial in the pathogenic phase of clinical pathogenic CRECs.

### Genome wide association analysis of CREC isolates

A total of 513 genes were found to be above the significance threshold (Bonferroni test, *p* < 0.01) between healthy and clinical populations. To further investigate these genes of differentially allele frequencies, including *bla*_NDM-5_ and genes related to its conserved genetic contexts, as well as *bla*_KPC_, which were previously found to be significantly different between clinical and non-clinical isolates, these genes were subjected to KEGG pathway enrichment analysis. We found that the genetic difference of clinical CRECs were mainly enriched in biosynthesis of siderophore group nonribosomal peptides pathways and metabolic pathways (*p* < 0.05) compared with those from non-clinical isolates (Fig. S7). On the other hand, the genetic difference of non-clinical isolates was mainly enriched in microbial metabolism pathways and quorum sensing pathways (*p* < 0.05) compared with those from clinical CRECs.

## Discussion

Currently, there are few studies on the prevalence of CREC in the normal flora of healthy people [13]. In this study, we conducted a systematic analysis of CREC isolates among healthy populations in different provinces of China between 2020 and 2022 and compare them with clinical CRECs with genomic analysis.

The carbapenemase genes in clinical CREC isolates that were collected from ICU patients in our previous study were mainly dominated by *bla*_KPC-2_ (53/116, 45.68%). However, unlike previous clinical CREC isolates, the most predominant carbapenemase gene was *bla*_NDM_ in healthy populations in this study (146/147, 99.32%) [6]. Asian countries, particularly China, are widely recognized as the primary reservoirs of the *bla*_NDM_ genes [35]. An earlier study reported that 80% of the isolates in Chinese patients from 2013 to 2015 carried *bla*_NDM-1_, with only 17.8% being *bla*_NDM-5_ [36]. However, the number of *bla*_NDM-5_-positive *E. coli* is much higher than that of *bla*_NDM-1_-positive *E. coli* in recent years, which may be attributed to the lower fitness cost of *bla*_NDM-5_ to host bacteria and its higher hydrolytic activity against carbapenems [37–39]. Tigecycline and polymyxin are important drugs for the clinical treatment of carbapenem-resistant Enterobacteriaceae infections [40]. However, the discovery of the *tet*(X) and *mcr-1* genes in CREC will further limit the selection of clinical drugs, potentially posing challenges to the effective management of CREC infections [41, 42].

The main ST of CRECs isolated from clinical patients was ST131 carrying the *bla*_KPC_ gene [2, 43]. The ST131 *E. coli* is a widely distributed exenteral-pathogenic *E. coli*, which has attracted extensive public attention due to its carriage of a variety of ARGs and VFs [44]. Interestingly, this particular ST was not detected in CREC isolates isolated from healthy people in the present study. The *bla*_NDM_-positive CRECs isolated from healthy individuals exhibited a more diverse range of STs, which may further expand the range of ARGs reservoirs. However, no dominant STs were detected in these *bla*_NDM_-positive CRECs, which is consistent with the results of previous studies [35]. This consistency underscores the significance of horizontal transmission as a pivotal route for the dissemination of carbapenemase genes in healthy individuals. This diversity in STs among healthy individuals suggests a broad dissemination of the *bla*_NDM_ genes in the normal flora, further emphasizing the importance of monitoring and controlling the spread of CREC in healthy populations.

Despite being isolated from various regions, some CRECs showed minimal genetic variation with only a few SNPs (*n* ≤ 20), indicating the possibility of clonal transmission. When these healthy individuals carrying CREC move in different regions, they pose a potential threat of transmitting resistance genes to other healthy individuals. Certain clinical and non-clinical CRECs clustered together in distinct phylogenetic groups with shared genetic backgrounds and identical VFs, suggesting a common origin. Moreover, some CRECs isolated from healthy people and clinical patients also displayed high genetic similarity (SNPs ≤ 20). The occurrence of this phenomenon may result from the exposure of healthy individuals to hospital environments containing multidrug-resistant strains. The majority of the *bla*_NDM_ genes are located on IncX3 type plasmids, consistent with previous clinical findings [6]. It has been demonstrated that IncX3 plasmids facilitate the transmission of *bla*_NDM-5_, and the *bla*_NDM-5_ gene in IncX3 plasmid can exist stably [45, 46]. The mobile genetic elements such as insertion sequences and plasmids are highly correlated with a variety of carbapenem resistance genes, and they play important roles in mediating the spread of carbapenem resistance genes [47]. This phenomenon may contribute to the widespread dissemination of carbapenem resistance genes among healthy populations.

The presence of class 1 integron serves as a crucial indicator of multidrug resistance. Our findings reveal a higher positive rate of *intI1* in both healthy individuals and clinical-origin CREC isolates, with no significant difference between the two groups (*p* > 0.05). Furthermore, all CRECs isolated from healthy individuals harbored multiple resistance genes, providing additional validation to this conclusion. This underscores the strong selection pressure on healthy humans for colonization by multidrug-resistant *E. coli*. Although there were no significant differences in the number of ARGs and insertion sequences between the CREC isolates from clinical patients and those from healthy people, the former carried more diverse types of VFs and plasmid replicon types (*p* < 0.05). Moreover, the distribution of VFs in clinical isolates of CREC isolates are more diverse. The chi-square test also showed that the CREC isolates from clinical sources carried many VFs with significant differences, such as *irp2*, *fyuA*, and *iucABCD*. This increase in VFs suggests that the isolates from clinical patients may be more pathogenic compared to those isolated from healthy individuals, which aligns with the observed clinical outcomes [48, 49].

KEGG enrichment analysis results also demonstrate that clinical CREC has higher invasiveness and energy metabolism-related genes, and the specific functional differences may be further analyzed and confirmed by results from meta-transcriptome and deep metagenome sequencing to determine the differences in functionality between patient and healthy gut microbiomes. Moreover, iron is an essential nutrient for the survival of both non-pathogenic and pathogenic *E. coli*. Pathogenic *E. coli* have evolved specific mechanisms to acquire iron from host cells, as iron is often sequestered by the host as part of the innate immune response [50]. Pathogenic *E. coli* mainly use siderophores, small molecules with high affinity for iron, to acquire this essential nutrient. Enterobactin and yersiniabactin which was found that it was significantly enriched in clinical CREC are among the different types of siderophores produced by pathogenic *E. coli* to scavenge iron from host cells [51, 52]. Once acquired, iron is used by the bacteria for a range of cellular processes, including energy production and DNA replication. Iron has also been implicated in the regulation of the type III secretion system [53], which was identified significantly enriched in clinical CREC, a major virulence factor in many pathogenic *E. coli* strains.

We acknowledge the limitations of the current study. Firstly, this was a retrospective study, and some information on the CREC isolates collected was missing, which could have affected the accuracy of our analysis. Additionally, it was not possible to determine the positive rate of CREC isolates in different regions due to the lack of complete data. Secondly, our study only focused on the molecular and genomic characteristics of CREC in the Chinese healthy populations, and thus, we lack a comprehensive understanding of CREC in healthy populations worldwide. Further research on a global scale is needed to fully comprehend the distribution and prevalence of CREC among healthy individuals in different regions.

## Conclusions

This study provides a comprehensive analysis of the prevalence and distribution characteristics of CRECs in healthy individuals across the country. The CREC isolates isolated from healthy individuals were distributed across a variety of STs, and these STs were generally associated with low virulence. However, there is a possibility of transmission of these isolates to clinical patients, rendering clinical treatment ineffective. The emergence of CRECs in healthy individuals from multiple provinces of China underscores the need for stringent monitoring and appropriate measures to mitigate the future threats posed by CREC strains.

### Supplementary Information

**Additional file 1: Fig. S1.** The network graph describing the co-occurrence pattern of ARGs with ISs. **Fig. S2.** Circular comparison of blaNDM-bearing IncHI2 and IncX3 plasmids. **Fig. S3.** Phylogenetic tree generated by core genome and genetic contexts alignment. **Fig. S4.** Intersection analysis of ARGs, VFs, insertion sequences and plasmid replicons among clinical and non-clinical sources. **Fig. S5.** Alpha diversity analysis of ARGs, VFs, plasmids replicons and insertion sequences among clinical and non-clinical sources. **Fig. S6.** Manhattan plot of differential genes between clinical and non-clinical sources. **Fig. S7.** KEGG enrichment analysis of clinical and non-clinical environments.

**Additional file 2: Table S1.** Basic information of 147 CREC isolates collected from healthy populations. **Table S2.** MIC result of 147 CREC isolates to 15 drugs. **Table S3.** The location of the carbapenem resistance genes. **Table S4.** SNPs among 260 CREC isolates collected from healthy populations. **Table S5.** Chi-square analysis of ARGs carried by CREC in healthy people and clinical sources. **Table S6.** Chi-square analysis of VFs carried by CREC in healthy people and clinical sources.

## Data Availability

Whole-genome sequence data generated in this study has been deposited in the National Center for Biotechnology Information under BioProject no. PRJNA996502. Two plasmids pC519-IncHI2 (NZ_OR395176) and p1505-1-IncX3 (NZ_OR395175) with nanopore sequencing have been submitted to NCBI database. All study data is included in the article and/or supporting information.
