# Supplementary material for: National genomic epidemiology investigation revealed the spread of carbapenem-resistant Escherichia coli in healthy populations and the impact on public health
Source: Genome Med. 2024 Apr 16;16:57. doi: 10.1186/s13073-024-01310-x (PMC11020349; doi:10.1186/s13073-024-01310-x)
Supplement: Supplementary file 1 — Additional file 1: Fig. S1. The network graph describing the co-occurrence pattern of ARGs with ISs. Fig. S2. Circular comparison of blaNDM-bearing IncHI2 and IncX3 plasmids. Fig. S3. Phylogenetic tree generated by core genome and genetic contexts alignment. Fig. S4. Intersection analysis of ARGs, VFs, insertion sequences and plasmid replicons among clinical and non-clinical sources. Fig. S5. Alpha diversity analysis of ARGs, VFs, plasmids replicons and insertion sequences among clinical and non-clinical sources. Fig. S6. Manhattan plot of differential genes between clinical and non-clinical sources. Fig. S7. KEGG enrichment analysis of clinical and non-clinical environments. [file 13073_2024_1310_MOESM1_ESM.docx]

**Supplementary appendix**

**Nationwide surveillance and genomic analysis of carbapenem-resistant *Escherichia coli* among healthy populations in China**

Yan Li^1＃^, Yanyan Zhang^2＃^, Xinran Sun^1^, Yuchen Wu^2^, Zelin Yan^2^, Xiaoyang Ju^2^, Yonglu Huang^2^, Hongwei Zhou^2^, Zhiqiang Wang^1,4^, Shaolin Wang^3^, Rong Zhang^2*^, Ruichao Li^1,4,5*^

^1^Jiangsu Co-Innovation Center for Prevention and Control of Important Animal Infectious Diseases and Zoonoses, College of Veterinary Medicine, Yangzhou University, Yangzhou, Jiangsu, P. R. China;

^2^Department of Clinical Laboratory, Second Affiliated Hospital of Zhejiang University, School of Medicine, Zhejiang, Hangzhou, P. R. China;

^3^College of Veterinary Medicine, China Agricultural University, Beijing, China;

^4^Jiangsu Key Lab of Zoonosis, Yangzhou University, Yangzhou, Jiangsu, P. R. China;

^5^Institute of Comparative Medicine, Yangzhou University, Yangzhou, Jiangsu, P. R. China.

**Keywords**

healthy populations; carbapenem resistance; *E. coli*; plasmids; diverse clones.

^＃^These authors contributed equally to this work.

Corresponding authors: Rong Zhang, Email: zhang-rong@zju.edu.cn. Second Affiliated Hospital of Zhejiang University, School of Medicine, Zhejiang, P. R. China. Ruichao Li, Email: rchl88@yzu.edu.cn. Yangzhou University, Yangzhou, Jiangsu, P. R. China;

**
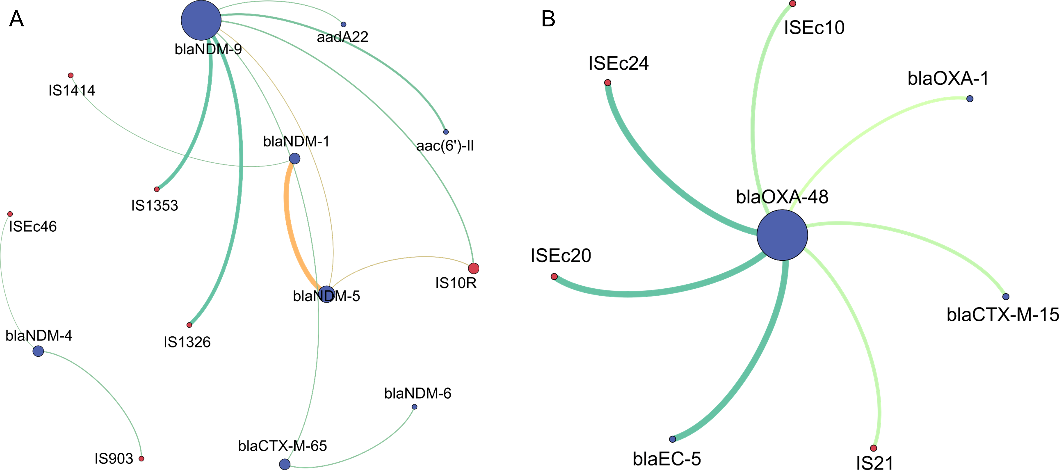
**

**Fig. S1. The network graph describing the co-occurrence pattern of ARGs with ISs.** (A) The correlation of *bla*_NDM_ with other ARGs and insertion sequences. (B) The correlation of *bla*_OXA-48_ with other ARGs and insertion sequences. Green colors and thicker lines represent stronger positive correlations. Blue dots represent resistance genes and red dots represent insertion sequences. The yellower the color represent the stronger the negative correlation.

**Fig. S2. Circular comparison of *bla*_NDM_-bearing IncHI2 and IncX3 plasmids.** (A) Circular comparison of *bla*_NDM_-bearing IncHI2 plasmids. Plasmid pC519-IncHI2 (NZ_OR395176) was used as the reference sequence. (B) Circular comparison of *bla*_NDM_-bearing IncX3 plasmids. Plasmid p1505-1-IncX3 (NZ_OR395175) was used as the reference sequence.

**
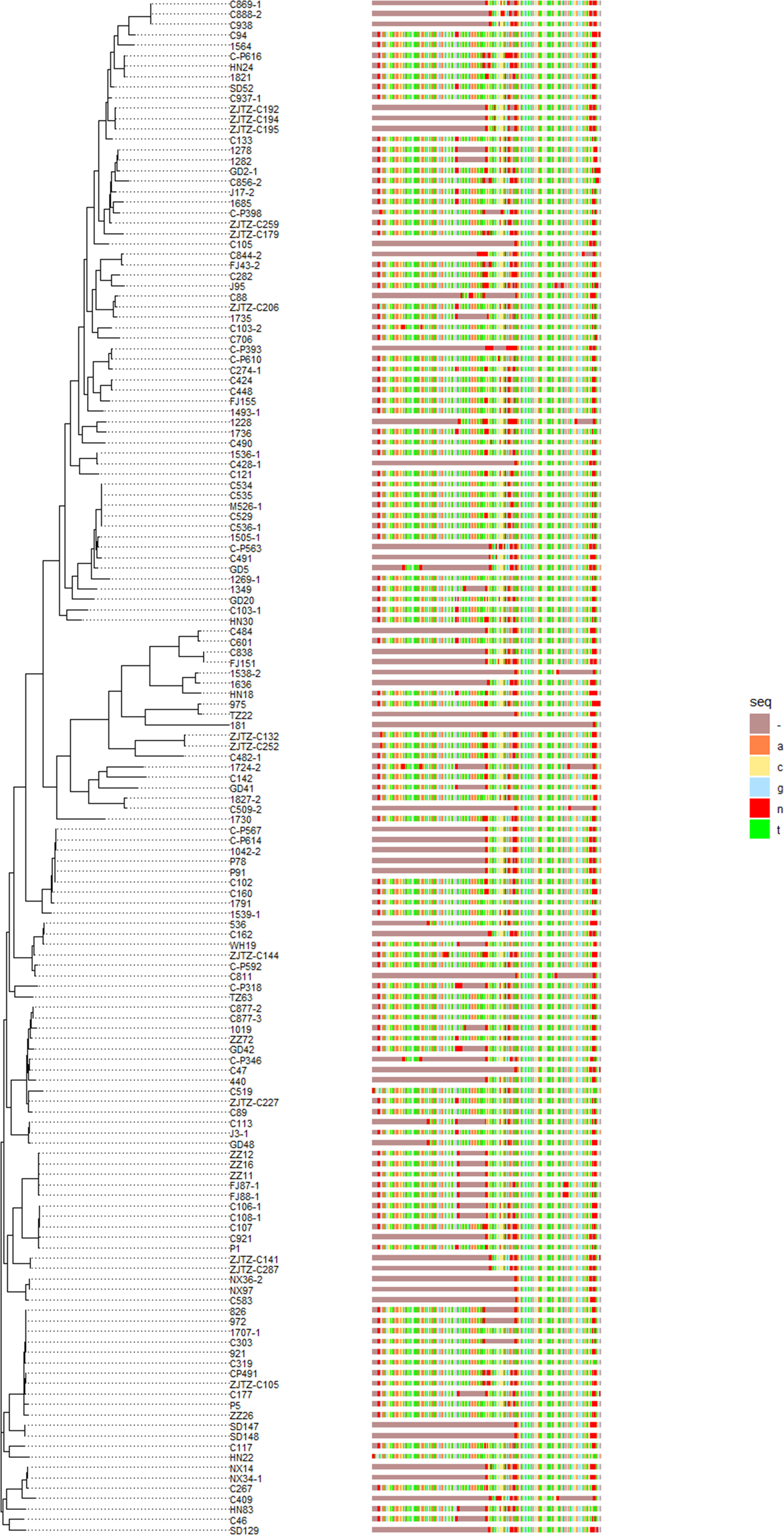
**

**Fig. S3. Phylogenetic tree generated by core genome and genetic context alignment.** The layout is organized with the core genome phylogenetic tree on the left and the multi-sequence alignment of the antibiotic resistance genetic context on the right.


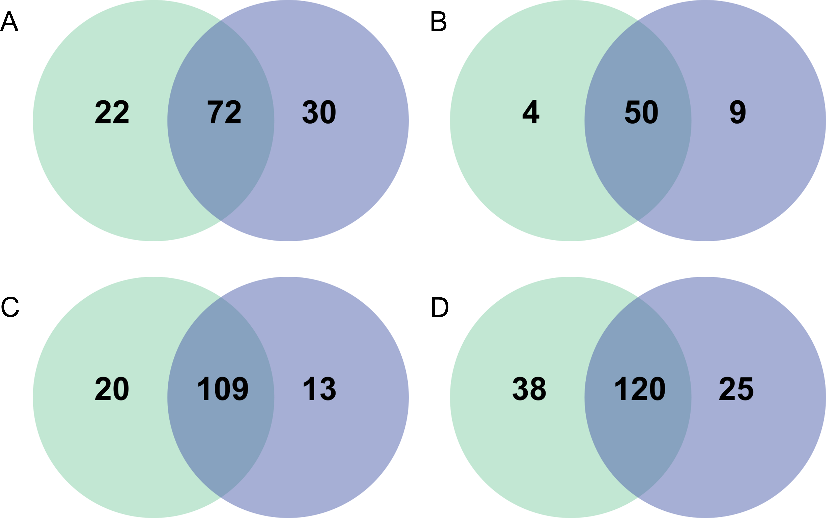


**Fig. S4. Intersection analysis of ARGs, VFs, insertion sequences and plasmid replicons among clinical and non-clinical sources.** (A) Venn plots of ARGs from different sources. (B) Venn plots of plasmid replicons from different sources. (C) Venn plots of insertion sequences from different sources. (D) Venn plots of VFs from different sources. Light green represents healthy person-derived CREC isolates and light blue represents patient-derived CREC isolates.


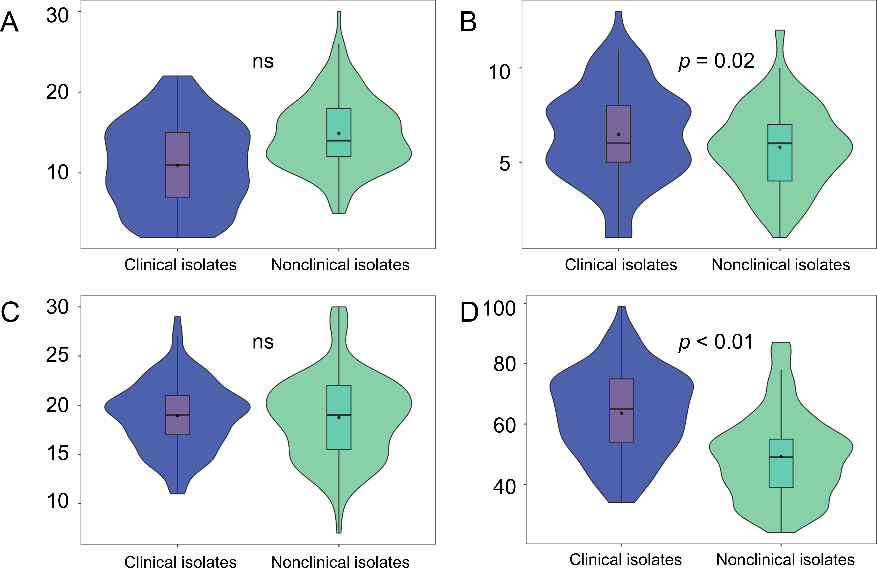


**Fig. S5. Alpha diversity analysis of ARGs, VFs, plasmids replicons and insertion sequences among clinical and non-clinical sources.** (A) Alpha diversity analysis of ARG numbers from different sources. (B) Alpha diversity analysis of plasmid replicons numbers from different sources. (C) Alpha diversity analysis of insertion sequences numbers from different sources. (D) Alpha diversity analysis of VFs numbers from different sources.


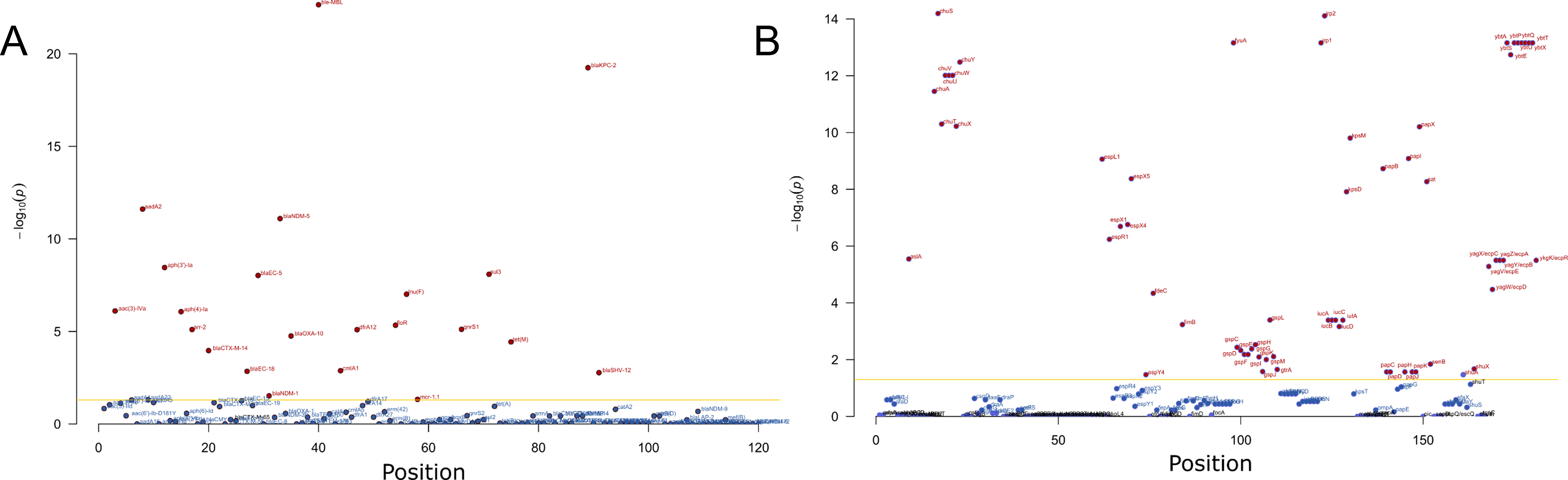


**Fig. S6. Manhattan plot of differential genes between clinical and non-clinical sources**. (A) Chi-square analysis of resistance genes. (B) Chi-square analysis of virulence genes. Genes with significant values above the threshold are labeled.


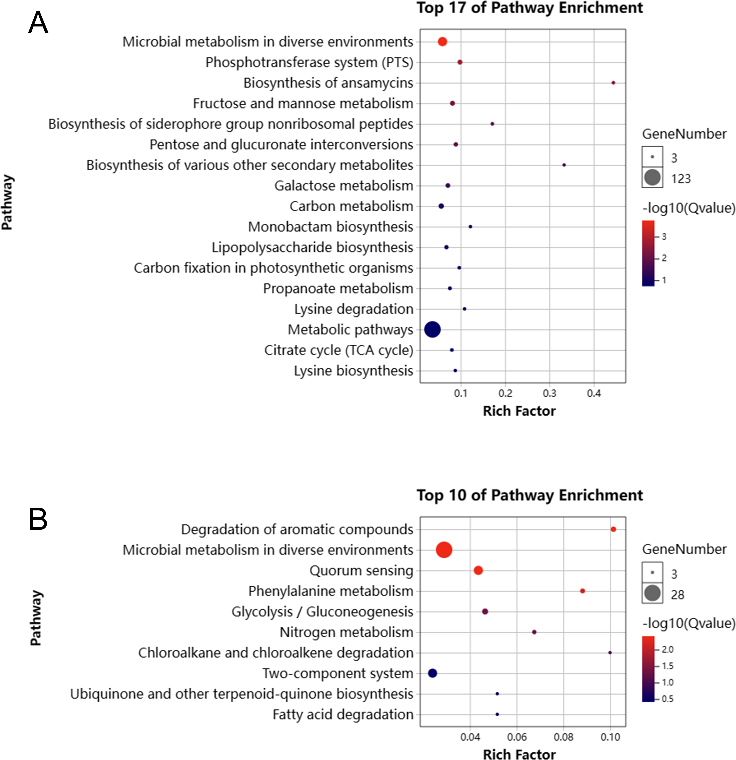


**Fig. S7. KEGG enrichment analysis of clinical and non-clinical strains.** (A) Pathways which were significant rich in clinical CRECs. (B) Pathways which were significant rich in non-clinical CRECs.
